# Development of an In Vivo Model for Eustachian Tube Dysfunction

**DOI:** 10.3390/bioengineering9070317

**Published:** 2022-07-15

**Authors:** Niels Oppel, Malena Ezzat, Philipp Krüger, Katharina Schmitt, Alexandra Napp, Friederike Pohl, Andre Bleich, Thomas Lenarz, Tobias Stein, Gerrit Paasche, Robert Schuon

**Affiliations:** 1 Department of Otorhinolaryngology, Hannover Medical School, Carl-Neuberg-Str. 1, 30625 Hannover, Germany; oppel.niels@mh-hannover.de (N.O.); ezzat.malena@mh-hannover.de (M.E.); schmitt.katharina@mh-hannover.de (K.S.); napp.alexandra@mh-hannover.de (A.N.); pohl.friederike@mh-hannover.de (F.P.); lenarz.thomas@mh-hannover.de (T.L.); schuon.robert@mh-hannover.de (R.S.); 2 bess pro GmbH, Gustav-Krone-Str. 7, 14167 Berlin, Germany; p.krueger@besspro.eu (P.K.); t.stein@bessgroup.com (T.S.); 3 Institute for Laboratory Animal Science and Central Animal Facility, Hannover Medical School, Carl-Neuberg-Str. 1, 30625 Hannover, Germany; bleich.andre@mh-hannover.de; 4 Cluster of Excellence Hearing4all, Hannover Medical School, Carl-Neuberg-Str. 1, 30625 Hannover, Germany

**Keywords:** otitis media with effusion, animal model, Eustachian tube dysfunction, hyaluronic acid, tympanometry, cone beam CT

## Abstract

Otitis media is often connected to Eustachian tube dysfunction (ETD). Until now, there was no large animal model available for the examination of new treatment methods such as stents for the Eustachian tube (ET). Thus, the aim of the study was to develop a method to reproducibly induce ETD by injection of fillers and without permanent closure of the ET. Tools for safe injection of hyaluronic acid (HA) in the surrounding of the ET were developed. In ex vivo experiments, HA mixed with Imeron^®^ was injected close to the nasopharyngeal orifice of the ET of blackface sheep. The established depot was visualized using cone beam computer tomography and magnetic resonance imaging, and stents could be placed into the ET. A reliable position of the HA depot was achieved. This method was transferred to in vivo, and middle ear ventilation was investigated by tympanometry. ETD was achieved with amounts of 2.5 mL HA or higher. None of the animals showed any sign of discomfort or complications. The induced ETD lasted for 3 to 13 (maximum observation period) weeks and was also combined with middle ear effusion. A model of ETD based on injection of HA next to the ET was successfully established and is now available to test novel treatment options for ET functionality.

## 1. Introduction

Otitis media with effusion (OME), where non-purulent fluid accumulates in the middle ear, is a leading cause of reversible hearing loss [1]. The Eustachian tube (ET) has a key role in the pathogenesis of otitis media (OM) [2]. It is the only connection between the middle ear and nasopharynx. It consists of an inelastic bony part, covering one-third of the full length of the ET, and an elastic cartilaginous part, covering the remaining two-thirds of the ET. The transition of these two parts forms a bottleneck: the isthmus [3]. The main functions of the ET include not only drainage and ventilation but also protection against infection and sound from the nasopharynx [4]. Chronic obstruction of the ET or ET dysfunction (ETD) results in inadequate equalization of the pressure in the middle ear, leading to prolonged negative pressure. The flat squamous epithelium of the middle ear develops into a highly prismatic epithelium with cilia and goblet cells due to the chronic negative pressure. Based on this epithelial change, more mucous secretion is produced, which can no longer be drained adequately [5]. This results in tympanic effusion, often followed by an inflammation of the middle ear and perforation of the tympanic membrane. The etiology of ET dysfunction comprises many different conditions, which should be identified by appropriate diagnostics.

The most common surgical approach to treat OM is the insertion of a tympanostomy tube into the tympanic membrane for ventilation and drainage of the tympanic cavity [6]. However, the middle ear is no longer separated from the external auditory canal, increasing the risk of infection and persistent perforation of the tympanic membrane [7].

Conservative treatments for ETD are the Valsalva maneuver for pressure equalization, nasal douching with saline solutions or nasal applications of decongestants, antihistamines and corticosteroids. Direct topical application of fluids into the ET could be an alternative [8]. Additionally, in the past, different tubes made of PVC, Silastic^®^ or gold were inserted over the open auditory cavity into the ET [9]. Another surgical approach was Eustachian laser tuboplasty, in which excessive tissue was removed to remove the obstruction [10]. The latest treatment is balloon dilation of the cartilaginous part of the ET to loosen adhesions and dilate the lumen [11,12].

For the purpose of further research, sheep have been proven to be an appropriate large animal model for middle and inner ear implantable hearing devices [13]. Additionally, Miller et al. [14] showed that the ET of blackface sheep matches the human ET in dimensions. Based on this study, stent implantation in the sheep as a new treatment option for ETD was successfully introduced [15]. The position of the stents in the ET of sheep were stable and the stents were well tolerated by the surrounding tissue. In terms of feasibility, the implantation was not performed in a dysfunctional ET and thus could not provide any proof of function.

To demonstrate function, an ETD model is needed. So far, described disease models of OME are using (I) irreversible methods like cauterization or ligation of the ET via a trans-neck or trans-oral approach [16,17], (II) functional obstruction via excision, transaction or transposition of the tensor veli palatini muscle [18], (III) injections of a chemical such as histamine solution through the tympanic membrane [19], (IV) the animal model Oxgr1 knock-out mice [20] or (V) induced infection using bacteria, such as Streptococcus pneumonia [17]. These animal models use rodents or rhesus macaques. Due to size limitations, stents developed for human application cannot be tested in these models. An attempt to use a large animal model was made by Pohl et al. [15], in which they used an approach to induce an aseptic OME with platelet-activating factor and prostaglandin E_2_ [21,22], but without success. Therefore, a large animal model to test treatments for ETD is still not available.

The objective of the current study was to develop and establish a large animal model for ETD. The aim was to provoke an aseptic mechanical obstruction of the ET based on a filler injection (Hyaluronic acid) into the surrounding tissue, which mimics the situation in human ETD.

Hyaluronic acid (HA) is a linear polysaccharide and occurs naturally in all vertebrates in the extracellular matrix of connective tissues and other tissues. In its natural form it has a half-life of 1–2 days in the tissue where it is degraded enzymatically. Cross-linking the HA generates a stabilized molecule that is water insoluble. Furthermore, it becomes hygroscopic. The half-life of stabilized HA is several months. For this reason, the HA remains stable in the tissue and will be resorbed more slowly [23,24]. HA is not only very well tolerated by the tissue, but in addition it is available with different degradation kinetics and physical properties. It is not only a standard substance in the esthetic surgery [23], but it is also very common in the clinical management of patulous ET [25].

Hyaluronidases are enzymes that degrade HA, occurring naturally in different forms. They are used in different application areas such as the management of complications associated with the injection of HA fillers [26]. This means that the applied HA can be dissolved more quickly by injecting hyaluronidase, if necessary.

Based on this, HA was selected as the filler for the augmentation of the tissue surrounding the ET. Through the mechanical pressure of the spatial augmentation, the ET would become obstructed and its function disabled.

To test a stent in a large animal model, there must be sufficient time between different anesthesia due to the necessary feeding restrictions. Therefore, the aim was to achieve a reliable ETD seven days after the intervention.

## 2. Materials and Methods

### 2.1. Ethic Statement

The in vivo experiments in this study were conducted in accordance with the German Animal Welfare Law and the European Directive 2010/63 and approved by the State Office for Consumer Protection and Food Safety, Dept. of Animal Welfare under the number 19/3255. The sheep were housed in the Central Animal Facility (CAF) of Hannover Medical School, and the experiments were performed with regards to the valid directives regarding accommodation, care and usage of experimental animals.

### 2.2. Hyaluronic Acid

Non-stabilised hyaluronic acid sodium salt from rooster (Sigma-Aldrich Chemie GmbH, Taufkirchen, Germany) was dissolved in isotonic saline solution 0.9% (Isotonic saline solution 0.9% ecoflac plus, B Braun Melsungen AG, Melsungen, Germany) at a concentration of 20 mg/mL and vortexed for 5 min.

Imeron^®^ 300 (Bracco imaging Deutschland GmbH, Konstanz, Germany) (0.2 mL/mL), a radiographic contrast enhancer, was added directly during the dissolution process if needed. This solution was then stored in a 15 mL falcon tube (Cellstar^®^ Tubes, 15 mL, Greiner Bio-One GmbH, Frickenhausen, Germany) for at least 12 h in a fridge and then transferred to 1 mL syringes (SOL-MTM, 1 mL, B Braun Melsungen AG).

As stabilised HA, the commercially available HA 20 mg/mL with lidocaine hydrochloride 3 mg/mL (Restylane^®^ LYFT Lidocaine 1 mL; GALDERMA, Lausanne, Switzerland) was used.

### 2.3. Injection Instrument

**Figure 1:**
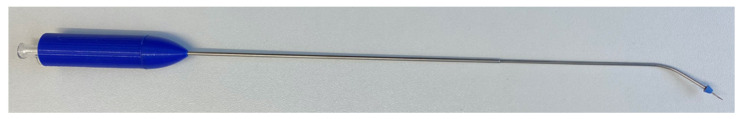
Injection instrument with the retractable cannula being pushed forward.

A specifically designed instrument (Figure 1) was used to inject HA into the mucosal tissue surrounding the ET. The outer tube, with a length of 250 mm and an outer diameter of 1.6 mm, was manufactured of stainless steel C15 (1.401). A blue-cone-shaped cover (made of styrene-ethylene-butylene-styrene) protects the tip of the instrument, affording atraumatic usage. The instrument has a 3D-printed polylactic acid handle with a length of 75 mm and is 16 mm in diameter. One side of the handle is flattened for handling and orientation reasons. The handle has a hollow end and a 2.5 mm wide and 20 mm deep cutout. Within this instrument there is a retractable cannula (23G, 0.6 mm in diameter) made of a hypotube and produced of stainless steel X5CrNi18-10 (1.4301) with a Luer-Lock connector. This cannula can be pushed 12 mm out of the atraumatic outer tube by placing the wing of the Luer-Lock connector inside the cutout and moving it forward. The metallic outer tube is bendable in the tip region. This allows modification of the cannula insertion angle to adapt to individual anatomical variations. In the course of this study, there have been different stages of development of this instrument; the one described here was the final version, which was used in 20 of 23 ex vivo tests and in all in vivo experiments.

### 2.4. Ex Vivo Experiments

This study was performed using 14 fresh frozen blackface sheep heads from the abattoir (Hencke Fleischwaren, Bad Bevensen, Germany) or the CAF. The heads were defrosted for at least 24 h at room temperature before performing the experiments.

#### 2.4.1. Endoscopic Procedure and HA Injection

A rigid endoscope (HOPKINS^®^ 70°, 4 mm in diameter, 30 cm in length; KARL STORZ SE & CO. KG, Tuttlingen, Germany) was attached to a telecam PAL (KARL STORZ) and connected to a Tele Pack Vet X LED RP 100 system (KARL STORZ) to observe the procedure.

The endoscope was introduced through the meatus nasi ventralis into the nasopharynx, contra laterally to the desired ET orifice. The 70° viewing angle was directed to the lateral side and moved forward closely following the inferior turbinate until the ET orifice was visualized. Then the endoscope was maneuvered laterally to position it anterior to the orifice and rotated to look sidewards to the ET orifice.

**Figure 2:**
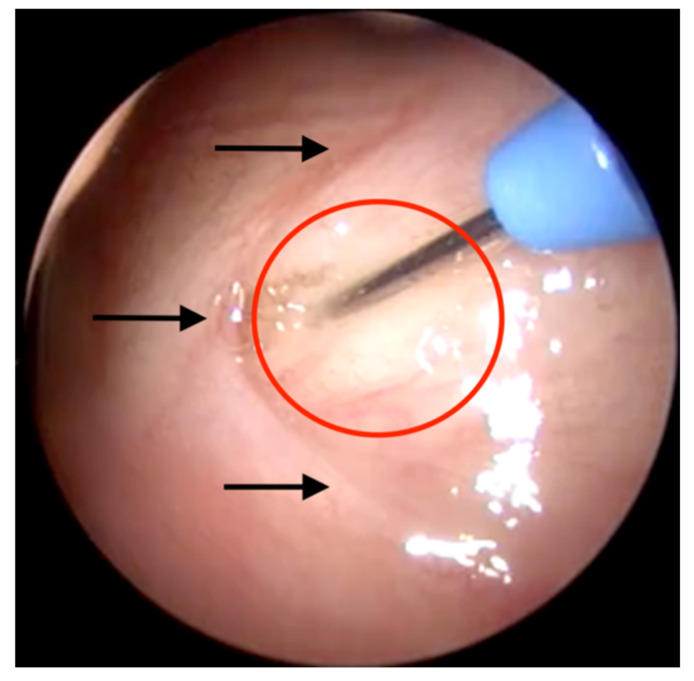
Endoscopic view of the pharyngeal orifice of the ET. The cannula is inserted in the target area (marked in red) in front of the crescent-shaped ET orifice (black arrows).

The cannula of the injection instrument was prefilled with HA. It was then inserted via the other nasal cavity into the nasopharynx by following the same approach. Thereafter, a slightly downward-facing rotational movement was performed until the tip of the instrument was visualised in the endoscope. It was then aligned medially into the target area just 1 to 2 mm centrally to the orifice (compare Figure 2). Once in place, the cannula was inserted laterally into the tissue of the ET (Figure 2). After securely placing the cannula in the mucosal tissue, the HA was injected slowly up to the desired amount. If the desired amount contained more than 1 mL, the connected syringe was replaced by a new, fully loaded syringe. During this procedure, the instrument stayed in position with the inserted cannula in the mucosal tissue. After the HA application, the cannula was withdrawn into the outer tube and the instrument was removed from the nasal cavity.

This procedure was applied to 23 ETs with variable amounts of stabilized and non-stabilized HA. An overview is provided in Table 1.

**Table 1:** Results of the ex vivo experiments.

ET	HA [mL]	Insertion Angle	Insertion Side	Outflow of HA	CBCT/MRI
**1**	0.3	n/a	n/a	No	
**2**	1	n/a	n/a	No	
**3**	0.3	n/a	n/a	No	
**4**	0.35	30°	Aligned	No	Good
**5**	0.35	30°	Aligned	No	Good
**6**	0.35	30°	Aligned	Yes	Good
**7**	0.4	30°	Aligned	No	Good
**8**	0.3	45°	Aligned	No	Good
**9**	0.3	45°	Aligned	No	Good
**10**	0.4	40°	Aligned	No	Good
**11**	0.4	40°	Central slightly behind the entrance	No	Too nuchal
**12**	0.3	40°	Aligned	Yes	Hardly visible
**13**	0.3	40°	Aligned	No	Good
**14**	0.3	40°	Aligned	No	Hardly visible
**15**	0.65	40°	Approx. 2 mm further rostral	Yes	Good with rostral bulge
**16**	0.3	50°	Central slightly behind the entrance	No	Good
**17**	0.4	40°	Aligned	No	Good with rostral bulge
**18a**	0.3	40°	Aligned	No	Good
**18b**	0.3 + 0.75	40°	Aligned	No	Good with rostral bulge
**19a**	0.5	40°	Aligned	No	Good
**19b**	0.5 + 0.5	40°	Aligned	Yes	Good with rostral bulge
**20**	0.2	40°	Approx. 4 mm further rostral	No	Just rostral of the ET
**21a**	0.4	40°	Aligned	No	Good
**21b**	0.4 + 0.2	40°	Approx. 2 mm further rostral	No	Good with a separate depot rostral
**22**	1.2 (s)	40°	Aligned	No	Good with rostral bulge (MRI)
**23**	0.5 (s)	40°	Aligned	No	Good (MRI)

n/a—not applicable (not documented); s—stabilized HA; aligned—position according to Figure 2; good—depot is located according Figure 3. In cases with a/b, the same ET was used for a second injection after a CBCT scan.

**Figure 3:**
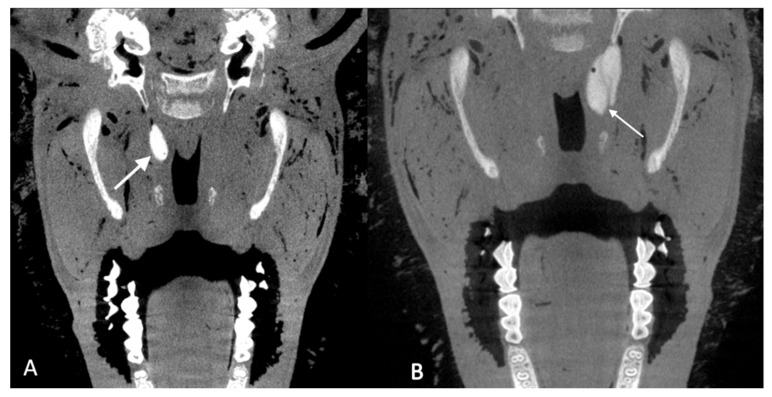
CBCT scans of cadaver heads after injection of 0.3 mL HA (**A**; case 13) or 1 mL HA (**B**; case 19b). The depots (white arrows) were visualized by addition of Imeron^®^ and were classified as good (**A**) and good with rostral bulge (**B**) (compare Table 1).

#### 2.4.2. Imaging

Before and after each step, a cone-beam computed tomography (CBCT) scan (XCat; Xoran^®^, voxel size 0.4 mm, Ann Arbor, MI, USA) was performed to visualize the distribution of the HA and Imeron^®^ mixture and to control the position of the instrument in the tissue.

Magnetic Resonance Imaging (MRI) was performed (MAGNETO Avanto 1.5T, Siemens Healthcare AG, Erlangen, Germany) using a T2-weighted 3D turbo spin echo protocol on one sheep head after the injection of stabilized HA.

#### 2.4.3. Feasibility of Stent Insertion

In total, 15 ETs were stented with a stent prototype for the ET (3–5 mm in diameter, 14 mm in length; bess medizintechnik, Berlin, Germany) after the injection of HA.

The stent was inserted using the same endoscopic procedure as described above. A stent application prototype instrument (bess Medizintechnik, Berlin, Germany) was introduced through the nasal cavity into the nasopharynx. Under endoscopic visualization, the instrument was inserted into the ET orifice up to a defined mark up on the instrument; the stent was then released, and the instrument was removed. The position and lumen of the stent were controlled by CBCT.

### 2.5. In Vivo Experiments

#### 2.5.1. Animals and Study Design

The in vivo experiment was performed with nine female, mature, healthy blackface sheep (provided by the CAF).

The experiment started by taming and training the sheep to tolerate the tympanometric measurement (for details, see below). After cleaning of the external ear canal (EAC) at week -1, tympanometric measurements were performed on two separate days to make sure that the middle ear pressure was in a physiological range. At week 0, the HA injection took place. After that, tympanometric measurements were then taken at least once a week during the entire experiment.

Five ETs were treated with non-stabilized HA and 17 with stabilized HA. Sheep without a proven ETD were re-introduced into the experiment after a minimum recovery period of 22 weeks, a veterinary examination and additional approval by the authorities (LAVES). As a result, nine animals were used for overall 22 in vivo HA applications.

The ETs treated with stabilized HA were divided into two groups. Group one, including eight cases, was monitored with tympanometry for one week after the injection and received a final endoscopic examination in week 1. Group two included nine cases and was controlled over 13 weeks using tympanometry. These cases underwent endoscopic examination in weeks 1, 7 and 13. During all endoscopic examinations of cases with stabilized HA, a subjective scoring of the protrusion of the depot (covered by mucosa) in the pharyngeal space was performed. It was divided into four categories; no protrusion, small, medium and strong, rated as 0, 1, 2 or 3, respectively.

#### 2.5.2. Cleaning the External Ear Canal

One week before the HA injection was performed, the EAC was cleaned mechanically under general anesthesia (GA) following the protocols of Pohl et al. 2017 [27].

After sedation with midazolam (0.2 mg/kg i.v.; Midazolam-ratiopharm 15 mg/3 mL, ratiopharm GmbH, Ulm, Germany), the GA was induced with propofol (5–10 mg/mL i.v.; Narcofol^®^ 10 mg/mL, CP-Pharma GmbH, Burgdorf, Germany). Anesthesia was maintained with isoflurane (1.5–2.0% end-tidal inhalation; Isufluran CP^®^ 1 mL/mL, CP-Pharma). Postoperative pain management was provided by carprofen (2 mg/kg i.v.; Carprosol^®^ 50 mg/mL, CP-Pharma) for each procedure under GA. All sheep were fixed in the thoracic-ventral position.

A looped probe was used under endoscopic control to remove excessive ear wax from the EAC (WEBER-LOCH ear loop size 1, KARL STORZ). If needed, the ear wax accumulations were dissolved with Otodine^®^ (main ingredients: propylenglycol, chlorhexidingluconat, pH 8 Tris-EDTA; aniMedica, Senden-Bössensell, Germany).

After the cleaning process, the EAC and the tympanic membrane were endoscopically examined for abnormalities. An inspection of the EAC and the tympanic membrane was performed each following GA.

The EAC was rinsed once a week with Otodine^®^ over the duration of the experiment to prevent accumulation of ear wax and ensure a free lumen.

#### 2.5.3. Tympanometry

To determine the middle ear pressure, tympanometric measurements were performed on conscious sheep with a commercial handheld tympanometer (Madsen Otoflex 100, GN Otometrics, Münster, Germany) using the method of Pohl et al. 2017 [27]. A special adapter [27] was used due to the longer EAC in sheep compared to humans. It consisted of a polyurethane tube (PUN-6 × 1, Festo AG & Co KG, Esslingen, Germany) attached to a connecting piece of a KimVent Microcuff Endotracheal Tube (Kimberley-Clark, Roswell, NM, USA). At the end of the tube, a red tip with a size of 10 mm (GN Otometrics) was used to hermetically seal the EAC.

The tympanometer was operated with a probe tone of 226 Hz, a pressure change (500–600 daPa/s, as fast as possible) from positive to negative pressure and a maximal range from −600 to 200 daPa. To achieve valid data, the measurement was carried out three times in succession for each ear in a quiet environment.

The tympanograms were classified into curve types according to Gelfand et al. [28]. Briefly, type A represents the physiologic middle ear status with equilibrated pressure in the tympanic cavity and, thus, an accurate working ET. A type B tympanogram has either no clear peak or a flat tracing. Various causes are described for a type B tympanogram such as a tympanic effusion, tympanic membrane perforation or a probe blocked by an accumulation of ear wax. These three causes can be differentiated by the measured ear canal volume (ECV). With OME, the ECV is in the physiological range; with a perforation of the tympanic membrane it is significantly increased, and in the case of the earwax it is significantly reduced. Type C tympanograms are shaped generally like a type A tympanogram, but the maximum compliance is shifted into the negative pressure area by more than 100 daPa. This indicates a non-physiological pressure in the middle ear and, therefore, a dysfunctional ET.

#### 2.5.4. Hyaluronic Acid Injection

The HA was injected under GA. Prior to the endoscopic intervention, a tip swab soaked with xylometazolinhydrochloride 10 mL (Otriven 0.1%, GlaxoSmithKline Consumer Healthcare GmbH & Co. KG, Munich, Germany) and lidocaine 5 mL (Xylocitin^®^-loc 2%, mibe GmbH, Brehna, Germany) was applied in both nostrils for 5 min to induce local anesthesia and to prevent bleeding. The injection itself was conducted according to the ex vivo experiments.

#### 2.5.5. Imaging

MRI was performed in one case, 13 weeks after the injection, with stabilised HA, using the same system and protocol as mentioned above.

#### 2.5.6. Health Score

During the entire experiment, animal health was checked regularly based on a health score [15] with small modifications (Table A1) and performed by veterinarians. Thus, separate categories were introduced for vocalization, activity and behavior/facial expression. These changes were made to speed up the detection of possible adverse clinical signs associated with the trial.

## 3. Results

### 3.1. Ex Vivo Experiments

HA was successfully injected into the tissue of 23 ETs, with three ETs having two injections. An overview of the results is provided in Table 1. The desired location for the injection was achieved in 18 cases. In cases 15, 20 and 21b, the needle was inserted further rostral of the ostium. In cases 11 and 16, the tip of the needle was inside the ET ostium, and in the first three cases, the injection side was not further specified.

The used angles of the tip of the instrument were in a range of 30° to 50°; in most cases, 40° was used. Larger angles were generally difficult to insert due to the limitation of space in the nasal cavity.

The used quantities of injected HA were between 0.2 mL and 1.2 mL. In some cases (cases 6, 13, 15, 19b), back flushing of HA out of the puncture was observed. Application was stopped in these cases. In general, applying 1 mL of HA or more was difficult. Here, the resistance during injection increased.

After injection of 0.3 mL HA, no optical change of the surrounding tissue was detected. With about 0.5 mL HA, the surrounding tissue protruded into the pharyngeal space, and with even higher amounts, pronounced bulging rostral of the ET ostium was observed.

The depots of HA were visible in CBCT. In 13 cases the depots were classified as good (Figure 3A), meaning the depot was, as intended, distributed close to the ET. Six depots showed a pronounced rostral bulge (Figure 3B). One depot was just rostral of the ET entrance (case 20), another depot appeared to be nuchal of the ET (case 11) and two depots were hardly visible in the CBCT scans (cases 12 and 14).

**Figure 4:**
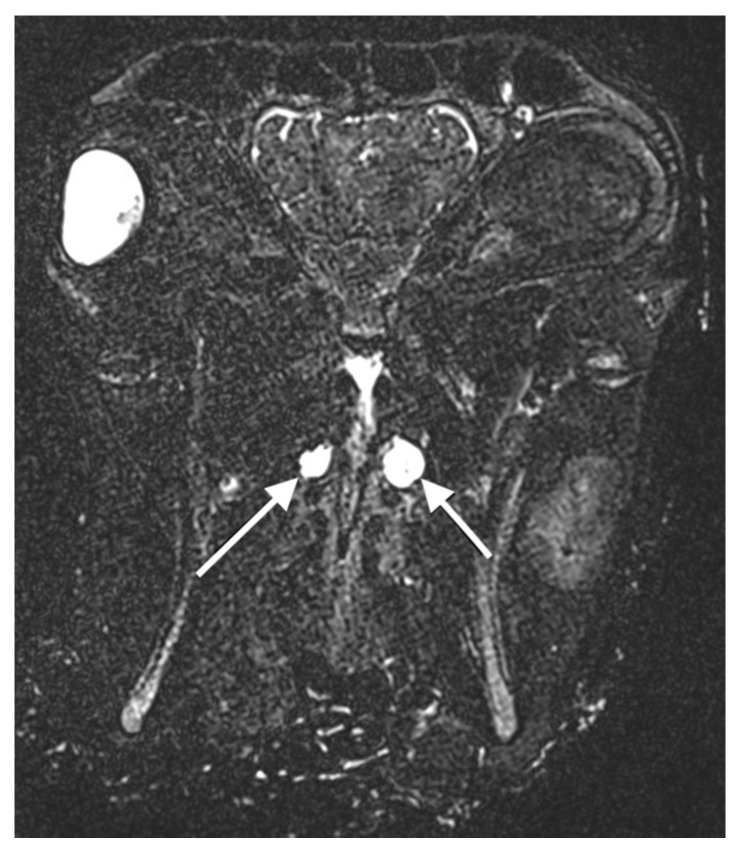
MRI images of a cadaver head (cases 22 and 23). The HA depots are marked by white arrows.

Using MRI (Figure 4), the shape and place of the depots were identical to the visualization with CBCT.

**Figure 5:**
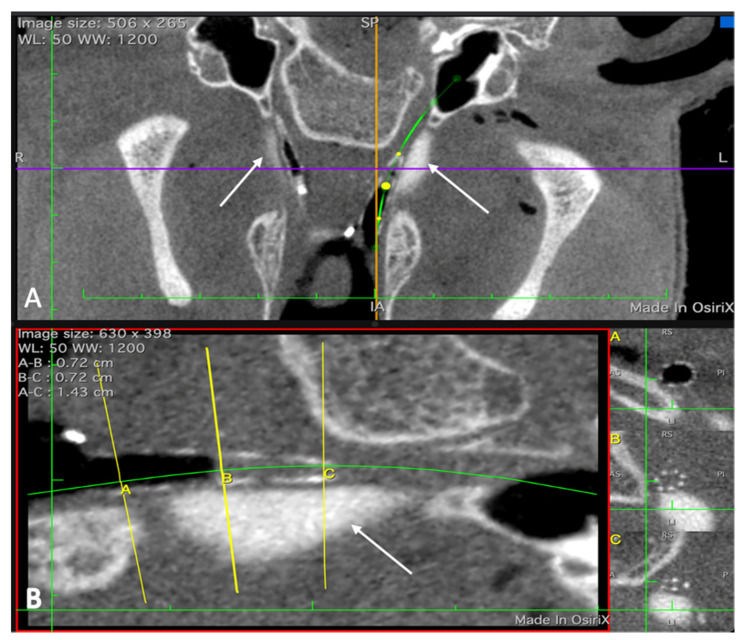
CBCT scan of a head with stent prototypes inserted into the ETs. (**A**): Both depots (cases 1 (left ET) and 2 (right ET)) are visible and marked by white arrows. Next to the depots, air filled stents can be seen. The green line indicates the course of the left ET. (**B**): Enlargement of one stented ET with the depot. The yellow lines (A; B; C) mark the position of the respective cross sections depicted on the right side.

A stent was successfully inserted into the augmented ET in 15 out of 15 experiments. Based on the CBCT images, the stent was at the desired place in the ET and unfolds completely (Figure 5).

### 3.2. In Vivo Experiments

All sheep had a physiological ET ostium, tympanic membrane and tympanometric measurements prior to the injection of HA. In all cases, the health score was 0 except for one sheep with score 1 for one day, 9 weeks after the injection, showing signs of acute bronchitis and an increase in body temperature to 41 °C.

#### 3.2.1. Injection of Non-Stabilized HA

The ET ostium was displayable in all cases. In one of the cases, a larger septum pharyngis was located in front of the ET ostium. A safe injection was not possible under direct vision in this case, and the injection was cancelled.

When using angles of more than 40° for the tip of the injection tool, the insertion of the instrument through the nose was difficult and quickly provoked bleeding in the nose during the insertion. This was also dependent on the size of the nostrils of the sheep.

Non-stabilized HA of increasing amounts from 0.3 mL up to 1 mL was used in five cases (Table 2). The used angles of the instrument were 45° or 50°. In four out of five cases, the injection was at the desired place. In case three, the needle slipped slightly behind the entrance of the ET just before penetration of the tissue. Therefore, a maximum of three insertions were needed to inject the desired amount of HA. In case four, some HA flushed out of the insertion site during the injection. A protrusion of the tissue into the pharyngeal space was slightly visible with an amount of 0.5 mL HA or higher.

In the following tympanometric measurements, no type C curve was measured with an amount of up to 0.9 mL HA (Table 3, cases 1–4). With an amount of 0.5 mL, a type B curve and a high ear canal volume were measured at days 7 and 9 (Table 3, case 2). During all other days, physiological type A curves were measured.

Using 1 mL HA, for the first time a type C curve was seen on the same day of the injection, one day after the injection and on day 14 (Table 3, case 5), but no stable ETD.

#### 3.2.2. Injection of Stabilized HA

Stabilized HA was injected in 17 cases with amounts from 1 mL up to 4.25 mL. An overview of the data is given in Table 4.

The bending angles used were in a range from 20° up to 50°. The insertions were atraumatic with the use of lower bending angles. In 14 cases, the needle could be inserted at the desired position. In two cases (6, 11), the point was missed slightly. For case 7, six different insertion points around the ET orifice were needed to safely inject an amount of 3 mL HA the first time.

In nine out of 17 cases, no HA flushed back out of the insertion site (Table 4). There seems to be a tendency for a higher risk of HA flushing back with larger amounts of fluid but also a reduction of appearance the more experience the team gained. In case 8, the desired amount of 2.5 mL HA could not be injected because the HA was flushing back out of the injection site.

**Figure 6:**
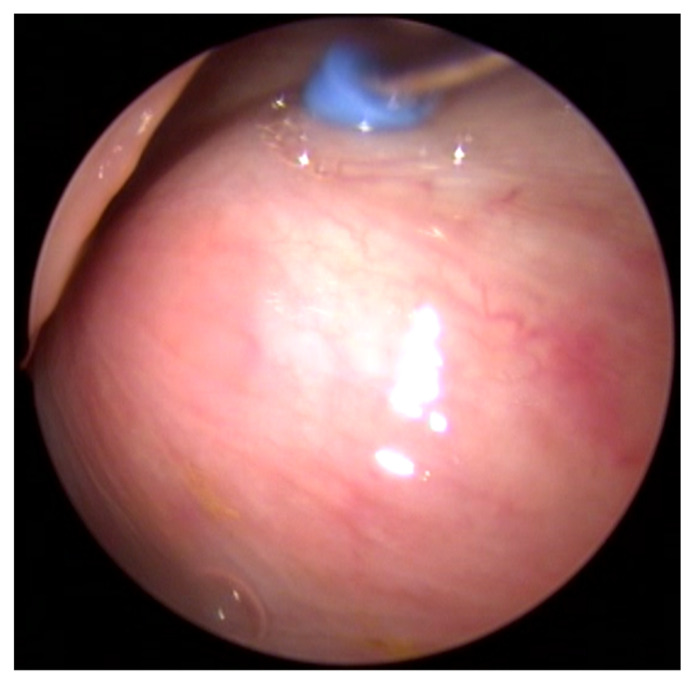
Endoscopic view of the ET of in vivo case 12 after injection of HA. A bulge is clearly visible in the ventro-rostral area of the tube (compare Figure 2). The insertion instrument is still in place.

**Figure 7:**
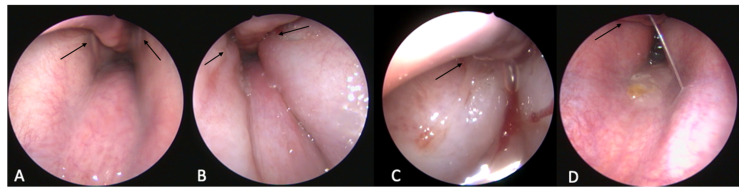
Endoscopic view of the nasopharynx of in vivo case 21/22. (**A**) Before HA injection; (**B**) after injecting the left tube (case 21, shown here on the right); (**C**) after bilateral injection; (**D**) on day 7. The ET orifices are marked by black arrows.

The surrounding tissue was visually augmented with a rostral bulge (Figure 6). This bulge was more and more increased with higher amounts of HA (compare Table 4). Additionally, the protrusion of the mucosa was increasingly concentrated in the rostral ventral area of the ET ostium (Figure 7B).

During the inspection one week after the intervention, an increase of the protrusion was visible in nine cases. Later, the change receded to varying degrees; in six out of seven cases, the protrusion was visible at week 7, and in six out of eight cases, this bulging was still apparent during the last inspections in week 13. A reduction in the amount of bulging over time was visible (Figure 7D). It has to be mentioned that in case 16, one week after injection of 3.5 mL HA, two mucosal lesions were seen on the bulge in front of the ET orifice. One was located medio rostral with a size of about 2 × 3–4 mm, sharply defined wound margins and purulent wound secretion. The other one was a little further nuchal, about 1 × 1 mm in size and approximately 0.5 mm deep. The surrounding mucosal tissue had shown signs of inflammation. There was no sign of HA coming out of the depot, and both lesions were completely healed in week 7 without any additional treatment.

**Figure 8:**
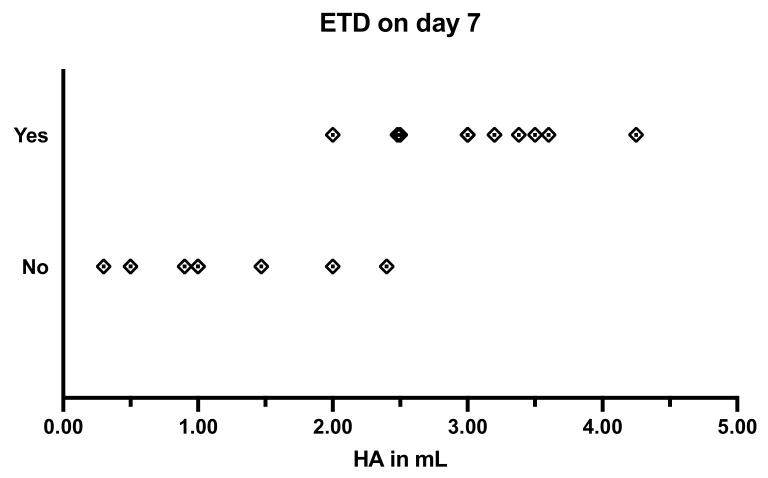
Success of the induction of ETD 7 days after injection of different amounts of HA.

When testing the ventilation of the middle ear by tympanometry, no major difference was found between 1 mL non-stabilized and 1 mL stabilized HA. An overview on the tympanometry results is given in Table 5. Injecting stabilized HA at amounts from 1 mL up to 2.4 mL induced mostly a pressure disturbance over a maximum of three days and a minimum of one day. The only exception was case 18, in which pathological tympanometric curves could be achieved for 7 days after the intervention with 2 mL HA. However, injecting an amount of 2.5 mL or more resulted in 11 out of 11 cases in a pathological curve at the target of 7 days after the injection (Figure 8). In ten cases, a type C curve was followed by a type B curve, indicating a middle ear effusion that developed. In two cases (10, 13), the ETD was stable throughout the longest study period of 13 weeks; in six cases, a type A curve was measured earlier. The shortest duration of ETD was 18 days, which was achieved with 3 mL in case 21.

In cases 10 and 13, in which the ventilation obstruction persisted until the end of the study period, the protrusion was categorized as small and nonexistent at the end of the observation period. By contrast, in cases 19, 17, 14, 8 and 7, medium was assigned once and the protrusion was otherwise small, and, in these cases, the ventilation disruption was no longer detectable.

#### 3.2.3. Tympanic Membrane

**Figure 9:**
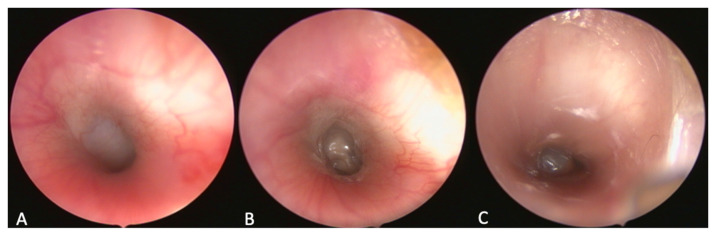
Endoscopic view of the tympanic membrane, (**A**) physiologic membrane (case 21, before the injection); (**B**) bulbed membrane (case 21, week 1); (**C**) fluid level in front of the membrane (case 10, week 1).

In seven cases, the tympanic membrane showed pathological signs of a bulging membrane after the intervention (Figure 9). In five of these cases (9, 11, 12, 13, 21), this bulging was observed at day 7 (including three cases out of the one-week follow-up group).

In case 14, bulging was only seen in week 7. Only in case 13, the tympanic membrane showed this change in all three examinations over 13 weeks. At day 7 in case 10, a fluid level was detected in front of the tympanic membrane (Figure 9). In the following examinations in weeks 7 and 13, the tympanic membrane was still bulging.

#### 3.2.4. Imaging

**Figure 10:**
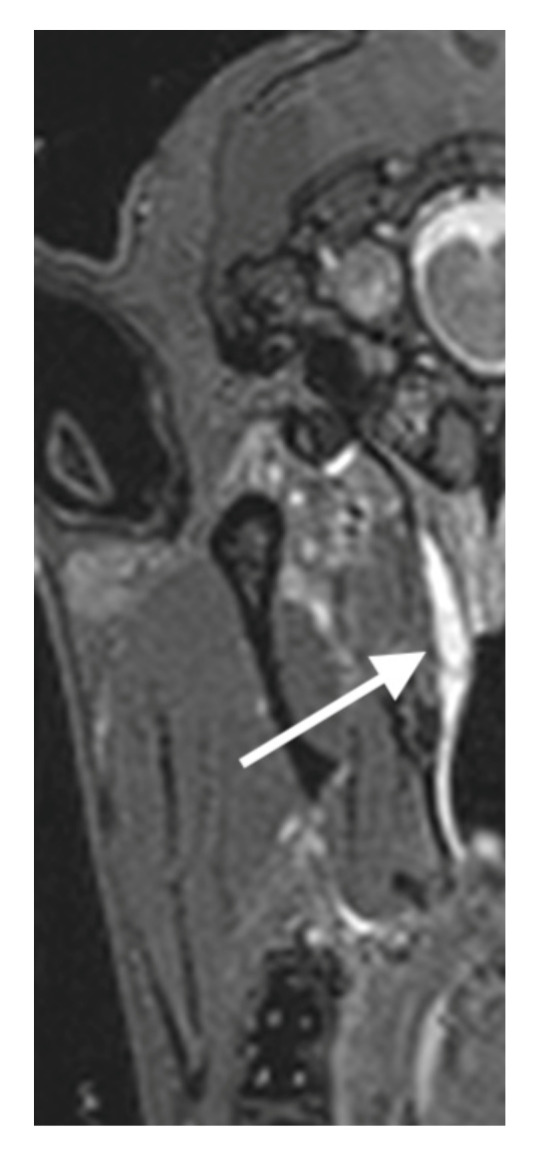
MRI image of case 21 after 13 weeks. The HA depot is marked by a white arrow.

In case 21, an MRI scan was performed after the 13-week follow-up period (Figure 10). This was the animal where the middle ear was ventilated again already 18 days after injection of the HA. The depot was still detectable in these images and is comparable in shape and position to the ex vivo CBCT and MRI images. The depot appears to be widened and extends further rostrally.

## 4. Discussion

New treatment methods for OM are needed, as there is no gold standard yet. In recent years, the treatment of ETD with a balloon catheter was established in a clinical setting [29], and the first experimental results with a stent in the ET were obtained in sheep [15]. Until now, there is no animal model of ETD available that also allows functional testing of treatments developed for human application.

With the instrument and procedure developed in the current study, the ET orifice could safely be reached during ex vivo testing, and the needle could be inserted at the desired location. Subsequently, a HA depot could reproducibly be applied. This procedure could be performed by one operator alone. The insertion depth of the cannula in this study was set to 12 mm, as this is half the length of the cartilaginous part of the ET of black face sheep [14], and the tubal cartilage is accompanying this entire length except for the first 1 or 2 mm [30]. As the ET narrows from the pharyngeal orifice towards the isthmus, generating a depot in the central region of the cartilaginous part should impede or at least disturb the natural function of the ET. An even deeper insertion of the cannula might potentially increase the risk of damage to the internal carotid artery and branches of the external carotid artery.

With the help of CBCT scans and the contrast agent Imeron^®^, the depot could be visualized in its position and shape in the ex vivo experiments. The position of the bean-shaped depot was parallel to the tube. At volumes greater than 0.4–0.5 mL, the depot progressively formed a bulge at its rostral end. This bulge increased with increasing amounts of HA. This increase was also clearly visible in the endoscopic examination. In two experiments, ex vivo and once after the end of the 13-week in vivo control period, an exemplary MRI image could be produced. Even after the 13 weeks, the HA is still clearly detectable. However, in this case, ETD was no longer detectable at this time. As the HA depot consists mainly of water [24], MRI imaging provides a good means to detect and investigate the position and shape of the generated HA depot. Unfortunately, for our study, we could only prove its applicability but could not use it on a regular basis.

The prototype stent was successfully inserted into the tube and fully deployed after the depot was placed. This forms the basis for testing stents in vivo once this animal model is established. In fact, only the stent could prove the position of the HA depot, as it is air filled after expansion, and the struts are also visible in CBCT. Without the stent, the ET is collapsed, and the position of the depot in respect to the ET cannot be evaluated in CBCT.

The procedure developed in the ex vivo experiments was then successfully transferred to the in vivo experiments. The HA could be injected into the tube using the same tool and procedure as in the ex vivo trials. However, only by increasing the amount to more than 2.5 mL and using stabilized HA was it possible to reliably induce an ETD. With an amount of around 2 mL, a brief disturbance of the tube function was detected. Based on this experiment, it is not possible to determine with certainty what led to the success (either the change to stabilized HA or the increase in quantity). After application of 1 mL, results with both HA were similar, so we might speculate that the increase in quantity was more important for the success. However, by using the stabilized HA and, thus, the longer half-life in the tissue, the goal of an ETD on day 7 was achieved. The efficacy of using Restylane^®^ and other stabilized HA fillers for augmentation in dermatology and aesthetic surgery has been reported to vary from 12 weeks to 16 months, with similar amounts of 0.5 mL to 3.9 mL of HA [31,32], depending on the localization of the application. This information is partially consistent with the results in this study. In two cases, ETD lasted for at least 13 weeks. However, much shorter periods were also observed.

A direct comparison of the results from the mentioned studies with the one conducted here is not possible. In these studies, the presence of a HA depot was investigated, whereas in our study, the HA depot must have certain properties to trigger an ETD. The insertion sites are different as well. In the studies mentioned above, HA is mainly used for augmentation of skin folds and enhancement of certain skin areas, whereas in the current study, a depot was positioned between two muscles and the tubal cartilage. It can be assumed that the two muscles, tensor veli palatini and levator veli palatini, which actively open the ET [33], are also active during masticatory movement and exert greater pressure as well as increased shear forces on the HA depot. This probably reduces the stability of the deposit and accelerates the degradation of the HA itself.

In the current study, there was no direct dependence between the injected amount of HA and the duration of the ETD. Thus, with an amount of 3 mL HA, a blockage of the tube for more than 18 days, until the end of the study period of a maximum of 87 days, was found (cases 7 and 13). With the highest injected amount of 3.65 mL, which was controlled over 13 weeks, an ETD of 62 days was achieved. Endoscopic grading of the protrusion of the depot at week 13 also did not correlate with tube blockage.

The position of the depot and its shape were controlled in the ex vivo experiments. With increasing amounts of HA, the shape of the depot changed from linear parallel to the ET to drop-like with an increasing protrusion into the pharyngeal space starting at 0.5 mL HA. Unfortunately, in vivo imaging was not available, but a similar protrusion rostral to the ET orifice was observed. This extended to the nuchal edge of the pterygoid bone. ETD was only reliably achieved with 2.5 mL or more HA in the depot, so we speculate that ETD was actually caused in the region of the pharyngeal orifice by the larger depot. Perhaps the lateral support by the bone is needed to exert enough pressure on the tube orifice to disrupt its function. In an inflammatory process of the ET it is more likely to be disturbed by the increased circulatory pressure due to the surrounding swelling tissue. This distinction needs to be considered in the evaluation of any treatment for ETD in this model.

An even more viscous and stable agent could also be successful with less than 2.5 mL. However, the advantages of HA (its tissue compatibility, the availability of different half-lives and the easy dissolution by hyaluronidase) were more important in this trial. By using the stabilized HA, the forces required to apply the HA were increased compared to the non-stabilized HA. This meant that another person was needed to maneuver the instrument and apply the HA. In the course of the study, it also became apparent that the depot could be placed most safely without HA flushing back when the cannula was inserted only once into the mucosa. The team achieved this with increasing reliability due to improved practice during the experiments.

Tympanometry is a suitable means of measuring the course of pressure in the middle ear of the sheep [27]. In the current study, a negative pressure in the middle ear could be measured first, starting shortly after the injection of HA and followed by a non-vibrating tympanic membrane (curve type B), which indicated a tympanic effusion. This suspected diagnosis could also be confirmed in the endoscopic examinations based on the bulged tympanic membrane. In one case, a tympanic membrane rupture could be diagnosed through tympanic effusion (Figure 9C). Based on these clinical symptoms, it can be concluded that the ETD triggered is very similar to ETD in humans. Therefore, this model offers opportunities for further research on the course, development and treatment of OM and pathologically related diseases.

Due to the difficulties in applying even 1 mL HA in the cadaver heads, in vivo experiments were started with a maximal deposition of 1 mL HA. No symptom or clinical stress was detected in any animal according to the health score. After these first experiments, the amount of HA was increased, still without any signs of discomfort. The one case of acute fever was presumably not directly related to the intervention. Due to the complication-free course of the induced ETD, there was no need to use hyaluronidase.

In summary, a model for the ETD in sheep was developed and is now available. This animal model is—in contrast to most other existing ETD models—directly comparable to humans in size and functionality of the ET [14]. In addition, the ET is not permanently closed by sutures or similar methods, which was used in other studies [16,34]. Thus, the function of the tube and the outflow of secretions in the middle ear is disturbed and the ventilation itself is not permanently blocked. Furthermore, the model was well tolerated by the sheep without any signs of discomfort, even with an obviously induced ETD. Therefore, treatment methods on the ET, such as a stent, balloon dilatation or similar, can still be performed and evaluated.

## 5. Conclusions

The procedure of generating a depot of HA next to the cartilaginous part of the ET was developed in the ex vivo experiments and was successfully transferred to the in vivo experiments. An amount of at least 2.5 mL of stabilized HA injected in the surrounding tissue of the ET was necessary to reliably induce an ETD on day 7 after injection. The ETD lasted between 3 and 13 weeks (maximum observation period) without influencing the animal’s behavior and health. Thus, an in vivo large animal model for ETD was successfully established. It is now available for further research regarding treatment of OM or ETD, especially if a size comparable to human is needed.

## Figures and Tables

**Table 2:** In vivo cases injected with non-stabilized HA in chronological order.

ET Case	HA [mL]	Bending Angle	Insertion Point	Insertions Needed	Outflow of HA	Protrusion
**1**	0.3	45°	Aligned	1	No	0
**2**	0.5	50°	Aligned	2	No	1
**3**	0.5	50°	Centrally behind the entrance	2	No	0
**4**	0.9	50°	Aligned	3	Yes	1
**5**	1	45°	Aligned	1	No	1

Aligned—position according to Figure 2; protrusion rating: 0—no protrusion, 1—small.

**Table 3:** Results of tympanometric measurements over the experimental period after injection of non-stabilized HA.

	Days and Respective Tympanometric Measurement Results																
Case 1 (0.3 mL HA)	−7	0	1	5	12	19	26	36	40	47	54	61	68	77			
	A	A	A	A	A	A	A	A	A	A	A	A	A	A			
Case 2 (0.5 mL HA)	−7	−5	1	3	7	9	14	17	21	31	35	42	49	56	63	72	77
	A	A	A	A	B*	B*	A	A	A	A	A	A	A	A	A	A	A
Case 3 (0.5 mL HA)	−6	−1	1	3	6	13	20	30	34	41	48	55	62	71	76	83	90
	A	A	A	A	A	A	A	A	A	A	A	A	A	A	A	A	A
	−7	−4	0	1	3	10	14	21	28	35	42	51	56	63	70	78	
Case 4 (0.9 mL HA)	A	A	A	A	A	A	A	A	A	A	A	A	A	A	A	A	
Case 5 (1.0 mL HA)	A	A	C	C	A	A	C	A	A	A	A	A	A	A	A	A	

A—physiological curve type A; B*—flat curve but ear canal volume greater than 9 mL, indicating a ruptured tympanic membrane; C—pathological curve type C (shifted into the negative pressure range). Note: the days of tympanometric measurements vary among cases and are listed with the cases.

**Table 4:** In vivo cases injected with stabilized HA in chronological order.

ET Case	Observation [Weeks]	HA [mL]	Bending Angle	Insertion Point	Insertions Needed	Outflow of HA	Protrusion	Protrusion w 1	Protrusion w 7	Protrusion w 13 (12)	TM w 1	TM w 7	TM w 13 (12)
**6**	13	1	50°	Slightly inside the ET	1	No	1	n/a	n/a	n/a	n/a	n/a	n/a
**7**	12	3	35°	Distributed at the entrance	6	Yes	3	3 (day 14)	n/a	1	Phys. (day 14)	n/a	Phys.
**8**	13	1.47	40°	Aligned	4	Yes	1	1	1	1	Phys.	Phys.	Phys.
**9**	1	2.48	40°	Aligned	2	Yes	2	1	n/a	n/a	Bulg.	n/a	n/a
**10**	13	3.38	25°	Aligned	1	No	3	3	1	1	Rupt.	Bulg.	Bulg.
**11**	1	4.25	25°	Dorsally aligned	1	Yes	3	3	n/a	n/a	Bulg.	n/a	n/a
**12**	1	3.5	20°	Aligned	1	Yes	2	3	n/a	n/a	Bulg.	n/a	n/a
**13**	13	3	20°	Aligned	1	No	2	3	0	0	Bulg.	Bulg.	Bulg.
**14**	13	3.65	20°	Aligned	3	Yes	2	3	1	1	Phys.	Bulg.	Phys.
**15**	1	3	30°	Aligned	1	No	2	3	n/a	n/a	Phys.	n/a	n/a
**16**	1	2.5	25°	Aligned	1	No	2	3	n/a	n/a	Phys.	n/a	n/a
**17**	13	2.4	25°	Aligned	1	No	3	3	1	1	Phys.	Phys.	Phys.
**18**	1	2	25°	Aligned	1	No	2	3	n/a	n/a	Phys.	n/a	n/a
**19**	13	2	30°	Aligned	1	No	2	3	2	2	Phys.	Phys.	Phys.
**20**	1	3	30°	Aligned	1	Yes	3	3	n/a	n/a	Phys.	n/a	n/a
**21**	13	3	40°	Aligned	1	Yes	3	2	2	0	Bulg.	Phys.	Phys.
**22**	1	3.2	40°	Aligned	1	No	3	2	n/a	n/a	Phys.	n/a	n/a

n/a—not applicable; Phys.—physiological; TM—tympanic membrane; w—week; Bulg.—bulged TM; Rupt.—fluid in front of TM (TM probably ruptured); aligned—location according Figure 2; protrusion rating: 0—no protrusion, 1—small, 2—medium, 3—strong.

**Table 5:** Results of tympanometric measurements over the experimental period after injection of stabilized HA.

	Days and Respective Tympanometric Mesurement Results																							
Case 6 (1.0 mL HA)	−7	−1	0	1	2	5	6	7	8	9	12	13	14	21	26	27	33	40	48	54	64	68	75	82
	A	A	A	A	C	A	C	A	A	A	A	A	A	A	C	C	A	A	A	A	A	A	A	A
Case 7 (3.0 mL HA)	−7	−3	0	1	2	3	4	5	←→		32	35	37	39	42	44	46	49	51	53	56	63	70	77
	A	A	C	C	C	C	C	B	B	B	B	A	C	C	A	A	A	B	C	A	A	C	B	A
	−10	−3	1	2	3	4	5	6	8	9	10	11	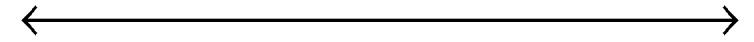											90
Case 8 (1.47 mL HA)	A	A	A	A	A	C	C	A	A	A	A	A	A	A	A	A	A	A	A	A	A	A	A	A
Case 9 (2.48 mL HA)	A	A	C	C	C	A	A	B																
Case 10 (3.38 mL HA)	A	A	C	C	C	C	B	B	B	B	B	B	B	B	B	B	B	B	B	B	B	B	B	B
Case 11 (4.25 mL HA)	A	A	C	C	C	C	B	B																
	−8	−1	1	2	3	4	5	6	8	9	10	11	12	13	14	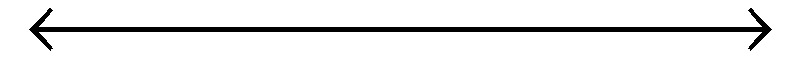								87
Case 12 (3.5 mL HA)	A	A	C	C	C	C	C	C																
Case 13 (3.0 mL HA)	A	A	C	C	C	C	C	C	C	C	C	C	C	B	B	B	B	B	B	B	B	B	B	B
	−8	−1	1	2	3	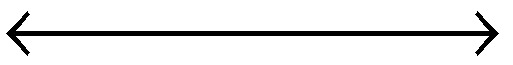					57	59	62	64	66	69	71	73	76	78	80	83	85	87
Case 14 (3.65 mL HA)	A	A	C	C	B	B	B	B	B	B	C	C	C	A	A	A	C	A	A	A	A	A	A	A
Case 15 (3.0 mL HA)	A	A	A	A	C	A	B	B																
	−7	−1	1	3	6	7	8	10	13	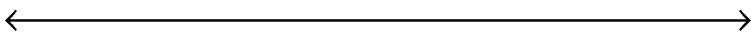														90
Case 16 (2.5 mL HA)	A	A	C	C	B	B																		
Case 17 (2.4 mL HA)	A	A	C	C	A	A	A	A	A	A	A	A	A	A	A	A	A	A	A	A	A	A	A	A
	−7	−1	1	3	6	7	8	←→		34	36	37	41	43	←→		64	66	69	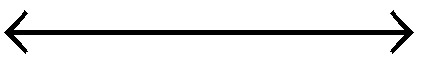				90
Case 18 (2.0 mL HA)	A	A	C	C	B	B																		
Case 19 (2.0 mL HA)	A	A	C	A	A	A	A	A	A	A	C	A	C	A	A	A	A	C	A	A	A	A	A	A
Case 20 (3.0 mL HA)	−14	−7	1	2	4	7																		
	A	A	B	B	B	B																		
	−4	−3	1	2	4	7	9	11	14	47	18	21	23	25	28	30	←→		74	77	79	←→		88
Case 21 (3.0 mL HA)	A	A	C	C	B	B	B	B	B	B	B	A	A	A	B	A	A	A	A	C	A	A	A	A
Case 22 (3.2 mL HA)	A	A	C	B	B	B																		

A—physiological curve type A; B—pathological flat curve type B; C—pathological curve type C (shifted into the negative pressure range). Note: the days of tympanometric measurements vary among cases and are listed with the cases.

## Data Availability

All the data that support the findings of this study are available on request from the corresponding author.

## Appendix A

**Table A1:** Health score as used in the current study. It was based on and modified from Pohl et al. [15].

Category	Observed Behavior	Score
Vocalization	Occasional communication with each other	0
	Occasional teeth grinding, sporadic snorting	1
	Frequent grinding of teeth, frequent snorting	2
	Moaning/sighing during expiration	3
Activity	Sleeping, quietly standing or lying down	0
	Frequent changes of position and attempts to stand up	1
	Restlessness (e.g., running around bleating)	2
Feed/water intake	Normal, normal rumination	0
	Only special food, moderate rumination	1
	No food intake, no rumination	2
Behavior/facial expression	Interested in the environment, searches in the straw, head is carried straight	0
	Depressed, dull, sporadic moderate head tilting and/or head shaking	1
	Flehming, absent staring, permanent head tilting and/or head shaking	2
Breathing rate	Up to 25 breaths/min	0
	25–30% increase above normal	0.5
	More than 50% increase above normal	1
Other abnormalities	None	0
	Temperature increase, serous nasal discharge	1
	Fever, purulent nasal discharge, bloody nasal discharge	2

Scoring was done daily for seven days after anesthesia, if inconspicuous thereafter according to the basic control interval every 2–3 days.
